# Emergency Department Doppler Assessment of a Central Retinal Artery Occlusion: Case Report

**DOI:** 10.5811/cpcem.1520

**Published:** 2024-03-26

**Authors:** Duncan McGuire, Robert Calleja, Eric Pai, Amit Bahl

**Affiliations:** * Ascension Providence Hospital, Department of Emergency Medicine, Southfield, Michigan; † Beaumont Health, Department of Emergency Medicine, Royal Oak, Michigan; ‡ Corewell Health, Department of Emergency Medicine, Royal Oak, Michigan

**Keywords:** *central retinal artery occlusion*, *point-of-care-ultrasound*, *retrobulbar spot sign*, *resistive index*, *case report*

## Abstract

**Introduction:**

Vision loss is a symptom found frequently in patients presenting to the emergency department (ED). Central retinal artery occlusion (CRAO) is an uncommon yet time-sensitive and critical cause of painless vision loss in which delayed diagnosis can lead to significant morbidity. Emergency medicine literature documents the ability to diagnose a CRAO using ultrasound by identifying the hyperechoic thrombus—coined the retrobulbar spot sign.

**Case Report:**

We present the case of a patient presenting with painless monocular vision loss for which CRAO was diagnosed in the ED using point-of-care ultrasound enhanced by the utilization of serial Doppler examinations as well as calculation of the central retinal artery resistive index.

**Conclusion:**

Despite the pre-existing literature on point-of-care ultrasound investigation of central retinal artery occlusion, there are no emergency medicine case reports describing serial examination of the central retinal artery by spectral Doppler or calculation of arterial resistive index to improve this evaluation and monitor progression of the pathology.

Population Health Research Capsule What do we already know about this clinical entity?

*Vision loss is a frequently encountered symptom in the ED.*

What makes this presentation of disease reportable?

*Point-of-care ultrasound (POCUS) can be rapidly performed in the ED to prevent delays in diagnosis of central retinal artery occlusions (CRAO), a potentially vision-threatening pathology.*

What is the major learning point?

*Serial POCUS examination in the ED can be used to both diagnose and monitor disease progression in CRAO.*

How might this improve emergency medicine practice?

*This case emphasizes the importance of POCUS in evaluating patients with painless vision loss to clinch the diagnosis and monitor progression of CRAO.*

## INTRODUCTION

Vision loss is a frequently encountered symptom resulting in numerous ED visits.^1^ While some ocular pathologies can be readily identified with limited history and evaluation, others require exhaustive approaches including dilated fundoscopic examination, specialized equipment, ophthalmology consultation, and even advanced imaging. Point-of-care ultrasound (POCUS) of the eye is a diagnostic modality that can be applied as an initial screening tool to help identify some higher risk diagnoses efficiently and accurately.^2^ This imaging modality allows for high-resolution evaluation of anterior and posterior chamber anatomy, as well as retrobulbar structures including the central retinal artery which travels within the optic nerve sheath.

Central retinal artery occlusion (CRAO) is the sudden blockage of the central retinal artery by occlusive thrombus or embolus that requires immediate evaluation and treatment at a comprehensive stroke center.^3^ As CRAO is an uncommon yet time-sensitive and critical cause of painless vision loss that may be difficult to distinguish from other benign causes, delayed diagnosis of CRAO is an unfortunate reality. The emergency medicine literature documents the ability to identify CRAO using POCUS by identifying a retrobulbar hyperechoic structure within the distal optic nerve sheath, representing central retinal artery thrombus (called the retrobulbar spot sign).^4^

There is, however, a paucity of literature describing the ED application of serial spectral Doppler examination as well as calculation of the central retinal artery resistive index (RI) to improve this evaluation and monitor pathology progression. We present the case of an ED using POCUS examination, enhanced by the utilization of serial Doppler examinations as well as RI calculation, to monitor disease progression.

## CASE REPORT

A 60-year-old female presented to a tertiary-care ED with a chief complaint of painless, right-sided monocular vision loss beginning 16 hours prior to arrival while she was eating dinner. At that time, she experienced an acute onset right-sided headache associated with painless vision loss in her right eye. The headache spontaneously resolved after three minutes; however, the persistence of visual deficits prompted her ED evaluation. Upon arrival to the ED, the patient endorsed a worsening right-sided superior quadrantanopia. She denied trauma, other neurologic deficits, headache recurrence, systemic symptoms, or a history of similar occurrence.

**Image 1.**
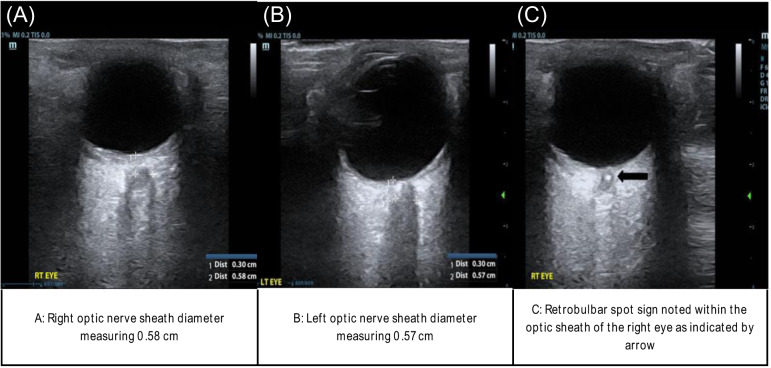
Point-of-care-ultrasound findings demonstrating normal right- (A) and left-sided (B) optic nerve sheath diameters, as well as an acute thrombus in the central retinal artery called the retrobulbar spot sign (C). *cm*, centimeter.

Gross ocular examination demonstrated pupils that were dilated to four millimeters (mm) bilaterally. The right pupil had delayed constriction after both direct and indirect light exposure compared to the left, consistent with a relative afferent pupillary defect. The patient’s right eye could perceive movement only out of the right upper quadrant field. Visual acuity was 20/200 oculus dexter (OD) and 20/25 oculus sinister (OS). Intraocular pressure was 16 millimeters of mercury (mm Hg) OD and 17 mm Hg OS. An ocular POCUS examination was performed by emergency physicians shortly after ED arrival. This demonstrated right optic nerve sheath diameter of 0.58 centimeters (cm) and left optic nerve sheath diameter of 0.57 cm (Image 1). There was no evidence of retinal detachment, posterior vitreous detachment, vitreous hemorrhage, retrobulbar hematoma, or lens dislocation. Upon closer examination of the optic nerve sheath, a hyperechoic signal at the distal aspect of the sheath, known as a retrobulbar spot sign, was appreciated (Image 1).

**Image 2.**
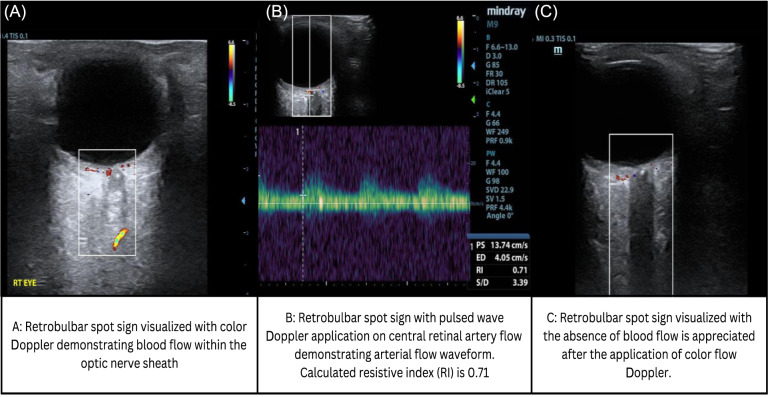
Central retinal artery with acute thrombus and color flow on color Doppler evaluation (A), central retinal artery with increased resistance to flow and an elevated resistive index (B), and absence of central retinal artery color Doppler flow upon repeat evaluation (C).

Color Doppler was applied to assess for central retinal arterial flow, and application of pulsed wave Doppler revealed an arterial flow pattern with an RI ([peak systolic velocity – end diastolic velocity]/peak systolic velocity]) calculated to be 0.71 (Image 2). After POCUS examination, the patient underwent additional imaging, ophthalmology, and laboratory evaluation. A non-contrast head computed tomography (CT) did not demonstrate acute intracranial pathology. Fundoscopic exam performed by ophthalmology demonstrated retinal whitening with superior macular sparing in the right eye with supero-nasal chorioretinal hyperpigmentation. The cup to disc ratio was 0.2∶1 bilaterally.

Upon repeat POCUS examination one hour later, there was no evident central retinal arterial flow indicating complete CRAO (Image 2). Due to this devascularization, a central retinal artery RI was unable to be recalculated. Because of the delayed ED presentation after symptom onset, the patient was not a candidate for tissue plasminogen activator (tPA); therefore, ocular massage therapy was initiated, and she was given a drop of brimonidine 0.1% ocular solution in the affected eye. Anterior chamber decompression was recommended; however, the patient declined. Given the atypical headache associated with the patient’s vision loss, erythrocyte sedimentation rate (ESR) and C-reactive protein (CRP) were ordered in the ED and were elevated (ESR = 40 mm/hr [reference range = 0–20 mm/hr] and CRP 23.4 milligrams per liter [mg/L] [reference range = <3 mg/L]). In case the vision loss occurred secondary to temporal arteritis, the patient was started on high-dose solumedrol (250 mg every six hours for 12 doses followed by prednisone 80 mg daily.

Neurology was also consulted in the ED, and the patient was admitted to the hospital for further management. Her hospital stay included a complete evaluation for stroke and inflammatory pathologies. She was discharged four days later on steroid, brimonidine, and aspirin therapy. The patient was provided with instructions to follow up with neurology, ophthalmology, cardiology, and rheumatology for continued evaluation and management. On subsequent reevaluation one month following her CRAO, the patient’s vision had improved to 20/70 -3 OD with extreme enhanced corneal compensation and 20/30+2 OS. Ophthalmology advised her to continue brimonidine eye drops and maintain strict blood glucose, blood pressure, and lipid control.

## DISCUSSION

Central retinal artery occlusion should be a consideration in patients experiencing painless vision loss, and dilated fundoscopic examination is the gold standard diagnostic approach.^5^ Given the nuances of the procedure including availability of specialized equipment, time needed for dilation, and environmental factors, emergency physicians are limited in their ability to accurately and efficiently perform fundoscopic evaluations. As ophthalmology consultation is not always readily available in many EDs, identifying CRAO can be a diagnostic conundrum with inevitable delays. Central retinal artery occlusion is a time-sensitive diagnosis and results in damage to retinal cells in as little as 12–15 minutes, underscoring the importance of early detection.^5^ Fortunately, emergency physician exposure to ocular POCUS is rapidly increasing, with the American College of Emergency Physicians recognizing ocular ultrasound as one of 12 core emergency ultrasound applications.^6^

While both globe and retrobulbar structures can be readily identified using ocular POCUS, evaluation for CRAO is only sparsely described in emergency medicine case reports or series.^7^ Additionally, there are no emergency medicine accounts of serial color Doppler exams performed on patients with the diagnosis of CRAO enhanced by the calculation of an RI. There are several ultrasound findings that may be present in the ocular POCUS examination of a patient suffering from a CRAO, including the following: an absence of pulsatile central retinal artery blood flow using color and pulsed wave Doppler, an increased RI measured using pulsed wave Doppler, and the retrobulbar spot sign.^3^ The retrobulbar spot sign is a hyperechoic structure found posterior to the eye within the optic nerve sheath. It is postulated that this structure has a hyperechoic sonographic appearance because it is a calcified cholesterol and thrombin embolus lodged within the central retinal artery.^4^

A color Doppler gait can be applied to the optic nerve sheath, revealing pulsatile flow within the central retinal artery.^8^ Once this is identified, a pulsed wave gait can be laid over this arterial color pattern. This allows for the calculation of the central retinal artery’s RI, the amount of resistance to blood flow within a vessel. It is calculated by detecting the variation in peak systolic and end diastolic velocities. A normal central retinal artery RI is less than 0.7.^9^ Presumably, the RI would be elevated in CRAO, although additional research is warranted to evaluate for the efficacy of this modality in predicting accurate central retinal artery vascular resistance.^10^ Additionally, there may be an absence of central retinal artery Doppler flow if there is complete occlusion of the vessel.

Currently, orbital CT angiography or fluorescein angiography are the imaging modalities recommended when attempting to diagnose CRAO.^5^ Diffusion-weighted magnetic resonance imaging can also be performed to evaluate for this pathology and retinal anatomy.^11^ However, these tests are not without their flaws as they take time to perform, expose the patient to radiation and intravenous contrast, are expensive, and are susceptible to motion artifact. By using POCUS as an adjunct diagnostic modality in patients suspected to have this diagnosis, emergency physicians can clinch this vision-threatening diagnosis, thereby avoiding further delays in the diagnostic and therapeutic pathways.

## CONCLUSION

Within the ED, ocular point-of-care ultrasound in patients experiencing acute onset painless vision loss can enable physicians to rapidly diagnose and accelerate treatment of central retinal artery occlusion. Our case highlights a novel use of POCUS in diagnosing and monitoring progression of this critical entity by using serial examinations aided by the application of spectral Doppler as well as resistive index calculation.
